# Evidence-informed language: interpretation and impact on intentions to treat – results of an online survey of medical students and specialists in German-speaking countries

**DOI:** 10.1136/bmjopen-2023-082907

**Published:** 2025-02-07

**Authors:** Reinhard Griebenow, Justine Schmidt, Henrik Herrmann, Sven Benson

**Affiliations:** 1 European Cardiology Section Foundation, Cologne, Germany; 2 Institute for Medical Education, Centre for Translational Neuro- and Behavioral Sciences (C-TNBS), University Hospital Essen, Essen, Germany; 3 Committee for Training and Education Politics, Marburger Bund Germany, Berlin, Germany

**Keywords:** Clinical Decision-Making, MEDICAL EDUCATION & TRAINING, Clinical Reasoning

## Abstract

**Abstract:**

**Objectives:**

Currently, there is no generally accepted consensus on how to translate strength of evidence into language. Against this background, we here investigated how widely used verbal descriptors of evidence grades and clinical practice recommendations, respectively, are understood, interpreted and transferred into intentions to treat. We explored differences between medical specialists and undergraduate medical students.

**Design:**

Cross-sectional, anonymous online survey. Assessment was based on publicly available proposals for wording to characterise strength of evidence from randomised versus non-randomised trials and of clinical practice recommendations, respectively.

**Setting:**

The online survey was conducted between September 2021 and March 2022 and promoted by several professional organisations in German-speaking European countries (Germany, Austria and Switzerland).

**Participants:**

Medical students, trainees and medical specialist (open to all medical specialties).

**Outcome:**

The survey was composed of two sections: (1) Aim of the first survey section was to assess if the linguistic differentiation between results from randomised versus non-randomised studies is correctly understood as put forward by the proponents. To this end, participants were asked to grade the relative weight of the expression for the results of a randomised trial versus two proposals for how to express the results of non-randomised studies. (2) Next, strong positive, weak positive, weak negative and strong negative clinical recommendations were presented, and participants were asked to answer in a forced-choice format if they would treat all/no patients, selected patients or only consider treatment. Additionally, the number of eligible patients who would be treated was assessed.

**Results:**

N=1081 physicians and N=539 medical students completed the survey. (1) Less than half of the participants (48.5%) interpreted use of ‘associated with’ as linguistic differentiation between results from randomised versus non-randomised trials in a sense as put forward by the proponents. However, use of the subjunctive mood (‘could’) resulted in 87.3% correct differentiations. (2) Even with only four types of clinical practice recommendations (positive/negative and strong/weak), interpretation and translation into intention to treat, respectively, showed a heterogeneous picture: while the presentation of a strong clinical recommendation led to largely congruent responses, the interpretation of weak recommendations showed a high variability, with no clear response pattern for intentions to treat. Responses from physicians and medical students were largely comparable.

**Conclusion:**

This study demonstrates limitations in the currently used linguistic expressions of strength of evidence and clinical practice recommendations and supports the need to prospectively test effects of language on intentions to treat prior to implementation of a certain wording. Study results cast doubt on that linguistic means alone will lead to optimally targeted intentions to treat.

**STRENGTHS AND LIMITATIONS OF THIS STUDY**

- Large sample from German-speaking countries composed of students and specialists, allowing to explore differences between novices and clinically experienced physicians.
- Testing of perception of verbal expressions of strength of evidence and intentions to treat based on evidence informed clinical practice recommendations in the same study population.
- The survey was conducted in German language and German-speaking participants only.
- The study population comprised a wide range of medical specialties but has not been representative for all German-speaking medical workforce.
- Findings from this cross-sectional online survey can offer only an approximation of how clinical recommendations are interpreted and transferred to intentions to treat in real-life clinical decision-making.

## Introduction

 Worldwide, the medical profession has claimed independence and professional autonomy to always ‘offer care in the patient’s best interests’.^1^,^3^

However, to achieve this aspiration, which even has constitutional status in some jurisdictions,^4^ one of the most important requirements is keeping medical knowledge up to date. But in the face of an ever faster growing body of medical knowledge,^5^ the acquisition, evaluation and integration of knowledge have gone far beyond the capabilities of the individual physician.^6 7^ This likely explains why systematic reviews, meta-analyses, health technology assessments and clinical practice guidelines have not only grown in popularity within the medical profession but also increased in legal significance.^8^

However, making decisions dependent on third party ratings and/or recommendations necessitates a clear framework to preserve the integrity of evidence-based medical practice. Methodologists have agreed on a detailed framework to assess strength of evidence.^9^ Yet, despite the fact that verbal or written communication is most widely used to share information in medicine and has a well-documented impact on treatment expectations and medical decision-making,^10^,^12^ there is currently no universally accepted consensus on how to translate strength of evidence and strength of recommendations into language, and current proposals have not been validated yet.^13 14^ This online survey investigated how widely used verbal descriptors of evidence grades and clinical recommendations, respectively, are understood, interpreted and transferred into intentions to treat.

Specifically, the study aims were to test in the same study population:

1. Which of two proposals for the linguistic differentiation between results from randomised controlled trials versus non-randomised studies^13 14^ leads to the clearest differentiation in the perception of participants?
2. How clinical treatment recommendations typically used in clinical practice guidelines are interpreted and transferred into intentions to treat?

The online survey was conducted in medical students and specialists from German-speaking countries, allowing an exploration of perceptions and attitudes, respectively, not only in clinically experienced physicians, but also in novices.

## Methods

This convenience sample survey was conducted anonymously online using the online survey tool Limesurvey (LimeSurvey, Hamburg, Germany; http://www.limesurvey.org). Participants were informed in writing about the study aims and then gave their informed consent to participate. The online survey was composed of two sections assessing how evidence grades and clinical practice recommendations were understood, interpreted and transferred into intentions to treat. Sociodemographic data on sex/gender, phase of medical study (ie, preclinical stage, semesters 1–4; clinical stage, semesters 5–10; ‘practical year’ (internships, semesters 11–12) or vocational position (ie, assistant physician/intern; specialist/consultant) along with length of medical practice were collected. The full survey can be found in online supplemental file 1 (German original with English translation).

The survey was designed by the authors (authors Griebenow, Schmidt and Benson). Items were self-constructed based on the wording of published recommendations (see below) given that no validated measures for the purpose of this study were available. The survey was promoted in Germany, Austria and Switzerland via medical faculties, scientific societies, professional unions and professional self-governance bodies (eg, Chamber of Physicians) between 23 September 2021 and 19 March 2022.

### Survey section 1: understanding of evidence grades

The aim of the first section of the survey was to assess if the linguistic differentiation between results from randomised versus non-randomised studies is correctly understood as put forward by the proponents. To this end, participants were asked to grade the relative weight of the expression for the results of a randomised trial versus two proposals for how to express the results of non-randomised studies.

That a certain drug causes improved life expectancy has been set as reference to express causality derived from results of (a) randomised clinical trial(s) (option B below).

Participants then were asked about their interpretation of option A (use of ‘associated with’ as key term to classify results from non-randomised trials^13^) or option C (use of subjunctive mood to classify results from non-randomised trials^14^) in relation to option B (use of ‘causes’) to find out which of the options might better discriminate between results from randomised vrsus non-randomised trials in the perception of participants:

- A ‘Treatment with drug X is associated with improved life expectancy’.
- B ‘Treatment with drug X causes improved life expectancy’.
- C ‘Treatment with drug X could improve life expectancy’.

A total of eight possible combinations (A=B=C; A=B>C; A>B=C; etc) were offered as a forced choice question.

Choosing the following combinations has been classified as in favour of the assumption that option A (‘associated with’) offers a valid linguistic differentiation between results from randomised versus non-randomised trials:

 B>A>C (ie, ‘causes’>‘associated with’>‘could cause’).

 B>A=C (ie, ‘causes’>‘associated with’=‘could cause’).

Choosing the following combinations has been classified as in favour of the assumption that option C (‘could cause’) offers a valid linguistic differentiation between results from randomised versus non-randomised trials:

 A=B>C (‘associated with’=‘causes’>‘could cause’).

 B>A>C (‘causes’>‘associated with’>‘could cause’).

 B>A=C (‘causes’>‘associated with’=‘could cause’).

Choosing the following combinations has been classified as disapproving the assumption that option A offers a valid linguistic differentiation between results from randomised versus non-randomised trials:

 A=B=C (‘associated with’=‘causes’=‘could cause’).

 A=B>C (‘associated with’=‘causes’>‘could cause’).

 A>B=C (‘associated with’>‘causes’=‘could cause’).

 A>B>C (‘associated with’>‘causes’>‘could cause’).

 C>B=A (‘could cause’>‘causes’=‘associated with’).

Choosing the following combinations has been classified as disapproving the assumption that option C offers a valid linguistic differentiation between results from randomised versus non-randomised trials:

 A=B=C (‘associated with’=‘causes’=‘could cause’).

 A>B=C (‘associated with’>‘causes’=‘could cause’).

 C>B=A (‘could cause’>‘causes’=‘associated with’).

 C>B>A (‘could cause’>‘causes’>‘associated with’).

### Survey section 2: interpretation of clinical practice recommendations and transfer into intentions to treat

The second part of the survey aimed to assess how participants understand clinical treatment recommendations. We used a simple four-class scheme taken from the Grading of Recommendations Assessment, Development and Evaluation (GRADE) group, that is, strong positive/weak positive/weak negative/strong negative recommendation.^15^ To this end, we assessed how the four different recommendations would be transferred into daily medical practice. The four recommendations were presented in a fixed order (strong positive, weak positive, weak negative and strong negative).

For the strong positive and the weak positive recommendation, participants were asked using a forced-choice format if they would:

- Apply treatment in all eligible patients.
- Apply treatment only in particularly promising cases (‘selected patients’).
- Only consider treatment (for eligible patients).

For the strong negative and the weak negative recommendation, participants were asked if they would:

- Apply treatment in none of the suitable patients.
- Refrain from treatment only in particularly high-risk cases (‘selected patients’).
- Only consider treatment (for eligible patients).

To address how each of these four recommendations are transferred into intentions to treat, participants were also asked to answer the question: ‘Out of 100 patients who are eligible for this treatment, how many patients would you treat?’ for each of the four recommendations. Answers were collected on a digital sliding scale (ie, a Visual Analogue Scale ranging from 0=none of the eligible patients to 100=all of the eligible patients). Instead of using the sliding scale, participants could indicate that they felt unable to answer the question. Please note that the participants who indicated they would treat ‘all patients’, but marked 0% on the sliding scale (n=1 student, n=3 physicians) and/or would treat ‘no patient’, but marked 100% on the sliding scale (n=15 students and n=70 physicians), were excluded from the analysis due to obviously erroneous responses.

### Patient and public involvement

Patients and the public were not involved in this study.

### Statistical analysis

Data are presented as absolute numbers and percentages or as mean and SD, as indicated. Subgroups were compared using χ^2^ tests for categorical variables and non-parametric Mann-Whitney U tests for continuous variables. Non-parametric tests were used because Kolmogorov-Smirnow tests indicated that data were not normally distributed. Statistical tests were only performed in subgroups of >100 participants to ensure sufficient statistical power (ie, 1−β>0.90, α=0.05) for medium-sized effects (d=0.5).

We analysed the following prespecified subgroups:

- First, the groups of students and physicians were compared.
- Students in the preclinical stage, clinical stage and the practical year were also compared within the subgroups of students.
- Within the subgroups of physicians, comparisons according to length of medical practice (<5 years vs 5–10 years vs >10 years) were made to explore the putative impact of vocational experience.

Results from the Visual Analogue Scales were also analysed descriptively using violin plots to visualise the distribution of answers (with answer density, median, first and third quartiles). The level of significance was set at 0.05. We applied Bonferroni correction to account for multiple testing as indicated in the results. SPSS V.27 was used for the analyses. The violin plots were created using GraphPad Prism (V.9). Power calculation was conducted with GPower V.3.1.9.7 (G*Power).

## Results

N=1643 submitted the survey. Only full data sets were included in the analysis (N=1621). Since one student indicated that they were not studying medicine, the final study population consisted of 1620 persons. Overall, 539 students and 1081 physicians participated, of which 742 (45.8%) were male, 871 (53.8%) female and 7 (0.4%) other. Sociodemographic characteristics are shown in table 1 and online supplemental table 1.

**Table 1:** Demographic data

Characteristics	N (%)
Total survey participants	1620 (100)
Medical students	539 (33.3)*
Preclinical stage	190 (35.3)
Clinical stage	230 (42.7)
Practical year	119 (22.1)
Physicians	1081 (66.7)†
Specialists	944 (87.3)
Trainee	137 (12.7)
Duration of medical practice	
<5 years	333 (30.8)
5–10 years	186 (17.2)
>10 years	562 (52.0)
Gender	
Female	871 (53.8)
Male	742 (45.8)
Diverse	7 (0.4)

* pPercentages for subcategories of medical students use 539 as denominator.

† pPercentages for subcategories of approbated physicians use 1081 as denominator.

### Survey section 1: understanding of evidence grades

Regarding the linguistic differentiation of results from randomised versus non-randomised trials, 48.5% of all participants had chosen combinations in favour of the proposal from the Heart Group Journal Editors (‘associated with’), while 48.2% had chosen an unfavourable option (ratio of favourable to unfavourable responses: 1.0) (table 2).

**Table 2:** Distribution of responses by expressions used to report results of randomised versus non-randomised studies (survey part 1)

Grading of wording used to describe strength of evidence†	∑ students	∑ physicians	Full sample
‘associated w’=‘causes’=‘could cause’	6 (1.1)	11 (1.0)	17 (1.0)
‘associated w’=‘causes’>‘could cause’	231 (42.9)*	398 (36.8)*	629 (38.8)
‘associated w’>‘causes’=‘could cause’	3 (0.6)	9 (0.8)	12 (.7)
‘causes’>‘associated w’>‘could cause’	220 (40.8)*	499 (46.2)*	719 (44.4)
‘causes’>‘associated w’=‘could cause’	30 (5.6)	37 (3.4)	67 (4.1)
‘associated w’>‘causes’>‘could cause’	29 (5.4)	73 (6.8)	102 (6.3)
‘could cause’>‘causes’=‘associated w’	14 (2.6)	25 (2.3)	39 (2.4)
‘could cause’>‘causes’>‘associated w’	6 (1.1)	29 (2.7)	35 (2.2)

Note. Data are shown in N (%). All percentages refer to columns. Column differences (ie, differences between subgroups) were examined using Chi-χ2 test followed by Bonferroni-corrected post- hoc Z- tests for column proportions (for exact values, see text).

For differences within the subgroups of students and physicians, respectively, see Supplementary Table 2online supplemental table 2.

* asterisks indicate sSignificant differences between the groups of students and physicians, respectively (*p*p<0.05).

† Options were A: ‘Treatment with drug X is associated with improved life expectancy’; B: ‘Treatment with drug X causes improved life expectancy’; C: ‘Treatment with drug X could improve life expectancy’.

On the other hand, 87.3% of all participants had chosen combinations in favour of the Marburger Bund proposal (subjunctive mood: ‘could’), while 6.3% had chosen an unfavourable option (ratio of favourable to unfavourable responses: 13.9).

A small group of participants (5.6%) did not meet any of the expectations, that is, assumed ‘associated w’=‘causes’=‘could cause’ or ‘could cause’>‘associated w’ or ‘causes’ to be correct. When comparing responses from students and physicians, a significant difference emerged (χ^2^=15.4; p<0.05). Post hoc tests indicated that physicians more often considered the ‘causes’>‘associated w’>‘could cause’ option as correct (p<0.05), whereas students more often chose ‘associated w’=‘causes’>‘could cause’ as the correct option (p<0.05). Additional analyses within subgroups of students and physicians indicated that duration of medical study or length of professional practice did not have an effect on the responses (see online supplemental table 2).

### Survey section 2: interpretation of clinical practice recommendations and transfer into intentions to treat

**Figure 1:**
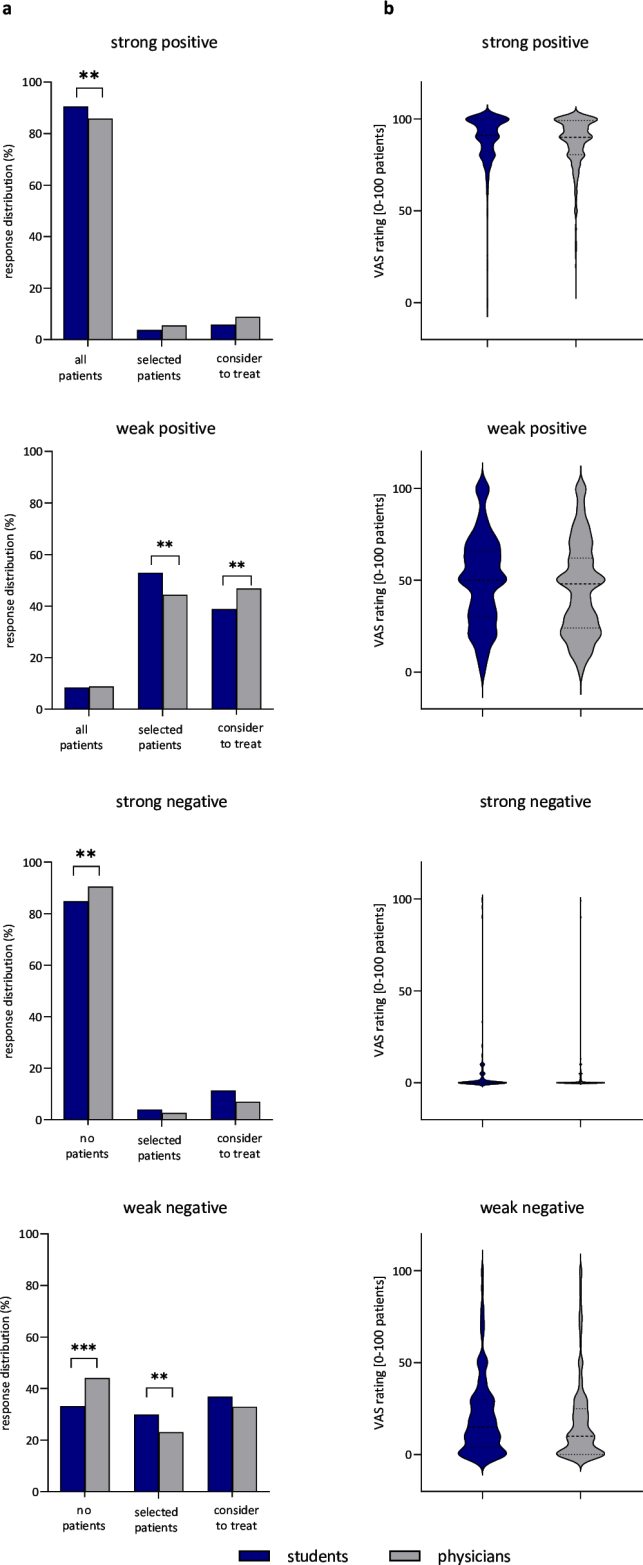
Results of survey section 2. (**a**) The percentages of participants who would treat all/no patients, selected patients or only consider to treat patients in case of a strong positive, weak positive, weak negative or strong negative recommendation, respectively. **p<0.01, ***p<0.001 (Bonferroni-corrected). (**b**) Participants were asked to quantify how many eligible patients they would use the treatment on a digital sliding scale ranging from 0 to 100 patients. Students are depicted in blue, physicians in grey.

In the case of a ‘strong positive’ recommendation, a high proportion of participants (87.3%) would apply this treatment in ‘all patients’. Physicians chose this option significantly less often than students (p<0.01) (table 3, online supplemental table 3, figure 1a).

**Table 3:** Distribution of responses for three response subcategories per clinical practice recommendation (survey part 2)

	∑ students	∑ physicians	Full sample
Strong positive recommendation			
Treat all eligible patients	488 (90.5)**	927 (85.8)**	1415 (87.3)
Treat selected patients	20 (3.7)	61 (5.5)	81 (4.9)
Consider to treat	31 (5.8)	97 (8.8)	128 (7.8)
Weak positive recommendation			
Treat all eligible patients	45 (8.3)	95 (8.8)	140 (8.6)
Treat selected patients	285 (52.9)**	480 (44.4)**	765 (47.2)
Consider to treat	209 (38.8)**	506 (46.8)**	715 (44.1)
Strong negative recommendation			
Treat none of eligible patients	457 (84.8)**	978 (90.5)**	1435 (88.6)
Treat selected patients	21 (3.9)	28 (2.6)	49 (3.0)
Consider to treat	61 (11.3)	75 (6.9)*	136 (8.4)
Weak negative recommendation			
Treat none of eligible patients	179 (33.2)***	476 (44)***	655 (40.4)
Treat selected patients	161 (29.9)**	249 (23.0)**	410 (25.3)
Consider to treat	199 (36.9)	356 (32.9)	555 (34.3)

Note. Data are shown in N (%). All percentages refer to columns. Column differences (ie, differences between subgroups) were examined using Chi-χ2 test followed by Bonferroni-corrected post- hoc Z- tests for column proportions (for exact values, see text).

For differences within the subgroups of students and physicians, respectively, see Supplementary Table 3online supplemental table 3.

* Asterisks indicate significant differences between the subgroups of students and physicians, respectively (**p<0.01, ***p<0.001).

To address how this choice was transferred into intentions to treat, participants were then asked to quantify the number of eligible patients in which they would use this treatment (digital sliding scale ranging from 0 to 100 patients). The mean number was 88.0±13.9 out of 100 eligible patients, with a slightly higher number in students (89.2±13.5) when compared with physicians (87.5±14.0; Z=−2.6, p<0.01, Ƞ^2^=0.005) (figure 1b). Out of all participants, 15.9% answered that they did not feel able to make a quantitative judgement (physicians less often than students; χ^2^=38.7, p<0.001) (online supplemental table 4).

We explored in addition, to how many of 100 eligible patients the participants would apply the treatment, depending on indication that they would treat all patients, selected patients, or only consider treatment (see online supplemental figure 1 for a detailed illustration). Briefly, even if respondents chose to treat ‘all’ patients, this translated into numbers of patients per individual physician in the range of 80% by just considering 1 SD below the mean. The roughly 13% of respondents who indicated they would treat selected patients or only consider treatment demonstrated a clear trend towards fewer patients receiving treatment. Due to large SD, this group showed not only substantial overlap with the ‘all patients’ group but also included numbers of patients well below the 50% level.

For the ‘weak positive’ recommendation, only 8.6% of the participants answered that they would apply this treatment in ‘all patients’. Students significantly more often chose the answer ‘apply treatment only in particularly promising cases’ (p<0.01), while physicians more often chose to ‘only consider treatment’ (p<0.01) (table 3, online supplemental figure 1).

When asked to quantify how many patients would receive the treatment, the mean number for the weak recommendation dropped to 46.4±24.7 out of 100 eligible patients, with a slightly higher number in students (49.7±23.9) compared with physicians (45±24.9; Z=−3.2, p<0.01, Ƞ^2^<0.01) (figure 1b). A rather high proportion of participants (25.6%) answered that they did not feel able to give a quantitative response. Physicians chose this option significantly less often than students (χ^2^=28.2, p<0.001) (online supplemental table 4).

The vast majority of physicians would treat 30%–80% of their patients based on a weak recommendation (online supplemental figure 1). Further verbal subclassification (‘all’, ‘selected’ and ‘only consider’) resulted in a trend towards less treatment but was not related to distinct differences in frequency of treatment decisions.

When a ‘weak negative’ recommendation was presented, 40.4% of participants (physicians more often than students, p<0.001) responded that they would ‘treat none of the patients’. Approximately a quarter of participants (25.3%; physicians less often than students, p<0.001) answered that they would ‘refrain from treatment only in particularly high-risk cases’ (table 3, figure 1a).

When asked how many patients would receive the treatment, the mean was 19.0±22.3 out of 100 eligible patients (physicians: 18.3±22.7; students 20.5±21.3; Z=−2.2, p<0.01, Ƞ^2^<0.01) (figure 1b). 26.3% of participants did not feel able to make a quantitative judgement (physicians significantly less often than students; χ^2^=19.9, p<0.001) (online supplemental table 4).

Up to 50% of patients might be treated by individual physicians despite a negative recommendation and further verbal subclassification was not related to differences in frequency of treatment decisions (online supplemental figure 1).

Finally, in the case of a ‘strong negative’ recommendation, 88.6% of participants would not apply this treatment to any of the eligible patients. Physicians chose this answer significantly more often than students (p<0.01) (table 3, figure 1a).

When asked how many patients would receive the treatment, the mean was 7.3±21.3 out of 100 eligible patients, with a negligible but significant difference between physicians (7.3±21.6) and students (7.2±20.5) (Z=−2.2, p<0.05, Ƞ^2^<0.01) (figure 1b). 16.4% of participants did not feel able to make a quantitative judgement using the sliding scale; physicians significantly less often than students (χ^2^=47.7, p<0.001) (online supplemental table 4).

Up to 25% of patients might still be treated by individual physicians who indicated that they would treat ‘none’ of their patients in the case of a strong negative recommendation (online supplemental figure 1). Further verbal subclassification shows no relation to differences in frequency of treatment decisions. For details regarding responses within the subgroups of students and physicians, see online supplemental tables 3 and 4.

## Discussion

This online survey offers insights on how verbal expressions of strength of evidence and clinical practice recommendations (based on strength of evidence), respectively, are understood, interpreted and transferred into intentions to treat the same population of medical professionals with backgrounds ranging from medical students to long-standing specialists. Data indicate that, depending on the term chosen, linguistic expressions of strength of evidence may result in the correct differentiation between evidence from randomised vrsus non-randomised trials in as low as 50% of the survey population, leading to the perception that all evidence is of equal strength. This needs to be taken into account in the interpretation of the survey results on how clinical practice recommendations are translated into clinical practice: Results support that both experienced physicians and students are basically willing to follow expert advice. However, the degree to which expert recommendations translate into quantitative decision-making, that is, how often a certain treatment is intended to be applied, primarily depends on the strength of evidence underlying the recommendations. Thus, ‘strong’ recommendations have shown to have a relatively consistent effect on intentions to treat, but ‘weak’ recommendations have been associated with high variance in intentions to treat. Moreover, a substantial number of participants, including experienced clinicians, were not able to ‘translate’ recommendations into quantitative terms, that is, a number of patients to be treated. Thus, this study demonstrates limitations of current clinical practice recommendations in offering guidance for clinical decision-making. The clarity of recommendations as well as their potential for benefit and/or harm needs to be critically discussed.

First, unclear linguistic expressions for the strength of evidence may, just as the misleading reporting of clinical trial results,^16^,^19^ lead to the misperception that all evidence is equally strong (as demonstrated in this study for the proposal of the Heart Group Journal editors). This would then counteract, for instance, the intentions of guideline writers to base the strength of recommendations on the strength of evidence. This will, as a consequence, have an impact on variability in intentions to treat, which is a particularly prominent finding in this study for ‘weak’ positive recommendations (see below).

According to the results of this study (conducted in German language), an acceptable distinction between, at least, the two principally distinct classes of evidence, that is, from randomised versus non-randomised trials, can be achieved best by using the subjunctive mood to describe data from non-randomised investigations. Further research will be needed to confirm this finding in languages other than German.

Second, previous research has shown that use of the word ‘must’ in clinical practice recommendations leads to the highest levels of obligation.^20^,^22^ However, the use of ‘must’ is usually avoided since it could imply a compulsory (eg, legal) obligation to perform a certain procedure when such an obligation does not, in fact, exist. Thus, ‘strong’ recommendations may not only result in lower levels of obligation–as demonstrated in this and other studies^20^,^22^–but also in lower attainment rates for highly recommended therapies, as demonstrated in health system research.^23^,^26^

Third, analogous to ‘strong positive’ recommendations, in this study, a ‘strong negative’ recommendation did not preclude that individual physicians might use such treatments in up to 25% of their patients. This corresponds to previous findings that the use of medical procedures discouraged by clinical recommendations is not uncommon.^27^,^29^ It might, therefore, be argued that in the case of negative recommendations, ‘strong negative’ should be replaced by ‘must not’ to more effectively avoid harm in patients.

Fourth, this study has revealed a dilemma in formulating concise clinical practice recommendations in the face of evidence that is not unequivocal.

Intentions to treat in response to ‘weak’ and particularly to ‘weak positive’ recommendations have shown a high variability, ranging from nearly never to almost always. This has been observed despite the use of an easy-to-grasp,^30^ four-class scheme of recommendations (adopted from GRADE group terminology^15^), and attempts to avoid distraction from strength of evidence by, in addition, providing patient characteristics (eg, in a clinical vignette), magnitude of clinical effect, etc. Thus, minimising the number of options for recommendations did not reduce the variability in intentions to treat observed for recommendations using a great variety of wordings.^20^,^22^

This may indicate that in the case of a ‘weak’ recommendation, intentions to treat are formed by a multifactorial complex decision process predominantly influenced by factors other than strength of evidence. Box 1 provides a (non-comprehensive) list of other factors leading to the formation of attitudes with potential or proven impact on intentions to treat.^31^,^70^

Box 1 **Factors with potential impact on physician attitude towards intentions to treat**

1. Professional attitude to ‘do something’.^31^,^33^
2. Differences in:
  - Tolerance for uncertainty leading to premature inclination to initiate or withhold action.^34^
  - Individual physician’s thresholds (eg, number needed to treat, number needed to harm) to start or withhold action in case of ‘weak’ recommendations.^35^
3. Financial issues, for example, related to personal^27^ or employer income.^36^
4. Legal issues, for example, related to liability thresholds (‘defensive medicine’^37^ or framework of the healthcare system^38^).
5. Doubts related to:
  - Robustness of trial findings.^1739^,^41^
  - Limitations in generalisability of evidence and hence of clinical practice recommendations.^42^,^44^
6. Credibility issues due to:
  - Conflicts of interest.^1645^,^51^
  - Intransparencies related to the body of evidence.^52 53^
  - Inappropriate interpretation of evidence.^16^,^19^
7. Scepticism towards validity of guideline recommendations.^1454^,^61^
8. Scepticism towards evidence-based medicine in general.^7 62^
9. Deficits in methodological competence.^63 64^
10. Priority of local consensus.^65^,^69^
11. Inertia to change clinical routine.^28^
12. Personal experience with patient preferences.^66 70^

Attitude formation obviously occurs early in a medical professional’s career since patterns of decisions are very similar between medical students and medical specialists.

In clinical practice, this means that even if it might be difficult to delineate an expected value or range of values for how often a certain procedure not backed by strong evidence may be applied, this will lead to underuse as well as overuse based on the same wording (and strength of evidence).

As far as quantitative effects have been reported,^27 36 45 65 66^ they are in an order of magnitude which corresponds to the variability of intentions seen in this study, particularly for a ‘weak positive’ recommendation.

Fifth, the additional use of pseudo-quantitative terms (such as ‘selected patients’ and ‘might be considered’) often used to characterise intentions to treat different from treating ‘all’ patients, at best, described a ‘bulge shift’ in the violin plots but had no added value in defining clearly separated patient groups. This corresponds to findings in the literature that introducing further explanatory items (eg, ‘strong positive’ means ‘a physician should act’) may even lead to confusion.^71^

Finally, it is a remarkable finding that up to a quarter of the respondents indicated being unable to translate the verbal terms describing clinical recommendations into a simple quantitative response, that is, the number of patients they would treat based on a specific recommendation. In other words, regularly used clinical recommendations obviously do not offer a good guide for clinical decision-making in a substantial number of physicians. Since similar numbers of clinicians and medical students have indicated not being able to transfer a recommendation into a quantitative response, this effect is probably not related to a lack of clinical experience or medical expertise. Although we cannot exclude that some participants refused to provide a quantitative answer due to a philosophy of medicine that considers each medical decision to be highly individualised, this cannot be the only explanation because response rates were related to the strength of evidence. The number of participants who did not provide quantitative responses substantially increased in the case of weak (vs strong) recommendations. This further supports the argument that weak recommendations are not well understood.

Our findings have several implications:

The powerful impact of verbal information on treatment-related expectations and decision-making in patients has been impressively demonstrated in placebo research and can even be used to improve treatment efficacy.^10^,^12^ However, exaggerated language can also be (mis)used to attract attention and influence decision-making in medicine.^72^ Control mechanisms such as journal guidance on language use, peer review or editorial procedures^1618 19 51 73^,^76^ have so far shown only limited efficacy in reducing misuse of language. Deficits in statistical competencies in medical professionals^63 64^ can further aggravate the detrimental effects of inappropriate language use. Our study indicates that flaws in expressing the strength of evidence can lead to misinterpretations by medical professionals, with comparable effects in students and medical specialists. Thus, a more precise and standardised approach to labelling evidence based on linguistic research is needed to maintain trust in evidence-based medicine and needs to be implemented across all fields of medical communication, ranging from classification of evidence to clinical practice recommendations, and from university lectures to continuing medical education.

Guideline writers have agreed that clinical practice recommendations should be ‘specific and unambiguous’.^77^ This seems to be difficult to achieve when substantial uncertainty remains with regard to the evidence base and, hence, recommendations are only ‘weak’. Thus, in quantitative terms, it will be difficult to define an expected value, or range of expected values, for the size of the patient cohort to whom a certain diagnostic or therapeutic procedure might be applied. This is further complicated by the fact that there seems to be no consensus among guideline writers on how often they expect the readers to act based on a certain recommendation wording.^22^ This study adds to the body of evidence indicating that linguistic means alone will not solve the issue.^20^,^22^ On the contrary, summary statement recommendations, as well as further pseudo-quantitative terms, have been shown to be associated with intentions leading to undertreatment and overtreatment and should, therefore, be avoided altogether. Alternatively, to become more ‘specific and unambiguous’, it seems reasonable to describe patient profiles in whom therapy might work best.^77 78^ This might enable physicians to better identify individual patients who might benefit most and reduce overtreatment or undertreatment.

In medical education, this approach may be further facilitated by procedural variables aiming to make strengths and weaknesses of the body of evidence more transparent, for example, by always reporting evidence from randomised trials first, before presenting data from non-randomised investigations, etc.^14^

Further research will be needed to elucidate whether non-verbal systems are better suited to reflect differences in strength of evidence.^79^,^81^

Health system research lacks consensus on what achievable attainment rates are, which have shown substantial variation even in the case of strongly recommended therapies.^15^,^18^ Results of this study indicate that interpretations of attainment rates probably fall short if they only relate to strength of recommendations and do not take into account other attitude-forming factors.

To clearly differentiate between legitimate professional autonomy and arbitrariness in medical decisions, physicians have committed to abide by professional standards.^1 3^ Our findings of non-negligible proportions of deviant decisions in the case of strong recommendations and high variability in the case of weak recommendations demonstrate that neither the type of linguistic recommendation (‘strong’, ‘weak’ or other) nor the actual average behaviour of the medical community, per se, can legitimately define the ‘standard’. This must instead take into account factors not assessed in this study, yet relevant to judge the legitimacy of decisions in the individual patient, including (among others) strength and applicability of evidence in the individual patient, considerations regarding individual benefit/harm, patient preferences and other considerations (eg, professional scrutiny, timing of decisions and availability issues).

According to the findings of this study, patients asking for a second opinion will get a deviating assessment based on the same strength of evidence and wording of the recommendations in a substantial number of cases based on strong recommendations. The likelihood of deviant advice further increases in the case of weak (particularly weak positive) recommendations. This may also lead to biased advice, if (in certain jurisdictions) courts would rely on the expert opinion of just one physician.

This study has several limitations: Findings and interpretation relate to German-speaking physicians only. Non-native speakers may have been lacking a thorough understanding of the questions as well as the options to respond to the questions.

Participants were invited to take part in the study through multiple medical associations. For this purpose, several large interdisciplinary professional organisations agreed to cooperate in this survey. However, it cannot be guaranteed that the responses are fully representative of the medical profession as a whole. Particularly the proportion of general practitioners is relatively low in this study, although they are often the ones who implement treatment recommendations in practice. However, it should be borne in mind that in Germany many specialists, especially internists, work as general practitioners, so the proportion of doctors working in general practice is likely to be higher than around 6.5%.

Testing both proposals in all combinations at the same time may have yielded results different from testing pairs only (eg, ‘associated with’<‘causes’ or = ‘causes’>‘could cause’.

Since published evidence does not suggest that offering a multitude of graded recommendations increases the discriminative power between recommendations,^13 14^ we have chosen to only offer three options to indicate how participants translate a recommendation into clinical practice. However, interpreting the often-used term ‘selected patients’ as ‘in most promising patients’ (or ‘not in high-risk patients only’ for negative recommendations) may not necessarily reflect the most preferred selection criteria of the participants.

This study has not been powered to detect further differences, for example, between medical specialties, etc.

It cannot be ruled out that answers were affected by unspecific interfering variables, for example, exhaustion or a distracting, turbulent surrounding.

Finally, findings from this cross-sectional online survey can offer only an approximation of how clinical recommendations are interpreted and transferred to intentions to treat in real-world clinical decision-making.

### Conclusions

In conclusion, this study has demonstrated current limitations in both linguistic expressions of strength of evidence and clinical practice recommendations. In the German language, differentiation between, at least, the two major classes of evidence (ie, data from randomised vs non-randomised studies) can be achieved best by using the subjunctive mood for findings from non-randomised investigations. Summary statements for clinical practice recommendations may work in the case of strong recommendations based on strong evidence, but for ‘weak’ recommendations (particularly weak positive recommendations) an alternative approach should be sought to make recommendations more explicit and thus reduce the huge variability in intentions to treat that is observed for this type of recommendation. In this regard, the additional use of pseudo-quantitative terms has been unrewarding. Finally, we have noted that a substantial number of respondents was not able to correlate linguistic terms with parameters of quantitative decision-making.

## supplementary material

10.1136/bmjopen-2023-082907 online supplemental file 1

## Data Availability

Data are available on reasonable request.
