# Supplementary material for: Evidence-informed language: interpretation and impact on intentions to treat – results of an online survey of medical students and specialists in German-speaking countries
Source: BMJ Open. 2025 Feb 7;15(2):e082907. doi: 10.1136/bmjopen-2023-082907 (PMC11808923; doi:10.1136/bmjopen-2023-082907)
Supplement: online supplemental file 1 [file bmjopen-15-2-s001.pdf]

Supplementary Materials

Evidence-informed language: interpretation and impact on intentions to treat - Results of an online survey of medical students and specialists in German-speaking countries

Reinhard Griebenow[1], Justine Schmidt[2], Henrik Herrmann[1,3], Sven Benson[2]

Supplementary Table 1: Medical specialties of physicians

	N (Specialist / trainee)	% (Specialist / trainee)
Internal medicine	284 / 49	26.3 / 4.5
Surgery	163 / 10	15.1 / 0.9
Anesthesia	117 / 16	10.8 / 1.5
Psychiatry and psychotherapy	67 / 6	6.2 / 0.6
Pediatrics	58 / 11	5.4 / 1.0
General medicine / Family medicine	50 / 20	4.6 / 1.9
Neurology	50 / 8	4.6 / 0.7
Gynecology and obstretrics	43 / 5	4.0 / 0.5
Radiology/ nuclear medicine	40 / 5	3.7 / 0.5
Psychosomatic medicine and psychotherapy	12 / 1	1.1 / 0.1
Others*	60 / 6	5.6 / 0.6
No. (%) of physicians	1081	100

*other specialties were otolaryngology, ophthalmology, occupational medicine, transfusion medicine, public health, human genetics, dermatology, pathology, microbiology, virology, laboratory medicine, physical and rehabilitative medicine

Supplementary Table 2: Distribution of responses by expressions used to report results of randomized vs. non-randomized studies (survey part one)

Grading of wording used to describe strength of evidence[ǂ]	Preclinical stage	Clinical stage	Practical year	∑ Students	<5 years medical practice	5-10 years medical practice	>10 years medical practice	∑ Physicians	Full sample
'associated w' = 'causes' = 'could cause'	3 (1.6)	1 (.4)	2 (1.7)	6 (1.1)	1 (.3)	1 (.5)	9 (1.6)	11 (1.0)	17 (1.0)
'associated w' = 'causes' > 'could cause'	78 (41.1)	96 (41.7)	57 (47.9)	**231 (42.9)***	131 (39.3)	77 (41.4)	190 (33.8)	**398 (36.8)***	629 (38.8)
'associated w' > 'causes' = 'could cause'	0 (0)	2 (.9)	1 (.8)	3 (0.6)	1 (.3)	2 (1.1)	6 (1.1)	9 (0.8)	12 (.7)
'causes' > 'associated w' > 'could cause'	78 (41.1)	95 (41.3)	47 (39.5)	**220 (40.8)***	164 (49.2)	80 (43.0)	255 (45.4)	**499 (46.2)***	719 (44.4)
'causes' > 'associated w' = 'could cause'	9 (4.7)	14 (6.1)	7 (5.9)	30 (5.6)	6 (1.8)	8 (4.3)	23 (4.1)	37 (3.4)	67 (4.1)
'associated w' > 'causes' > 'could cause'	10 (5.3)	15 (6.5)	4 (3.4)	29 (5.4)	17 (5.1)	10 (5.4)	46 (8.2)	73 (6.8)	102 (6.3)
'could cause' > 'causes' = 'associated w'	8 (4.2)	5 (2.2)	1 (.8)	14 (2.6)	6 (1.8)	4 (2.2)	15 (2.7)	25 (2.3)	39 (2.4)
'could cause' > 'causes' > 'associated w'	4 (2.1)	2 (.9)	0 (0)	6 (1.1)	7 (2.1)	4 (2.2)	18 (3.2)	29 (2.7)	35 (2.2)

Note. Data are shown in N (%). All percentages refer to columns. Column differences (i.e. differences between subgroups) were examined using Chi^2-test followed by Bonferroni-corrected post-hoc Z- tests for column proportions (for exact values, see text)

*asterisks indicate significant differences between the groups of students and physicians, respectively (*p<.05)

[ǂ]Options were A: 'Treatment with drug X is associated with improved life expectancy'; B: 'Treatment with drug X causes improved life expectancy';
C: 'Treatment with drug X could improve life expectancy'

Supplementary Table 3: Distribution of responses for three response subcategories per clinical practice recommendation (survey part two)

	Preclinical stage	Clinical stage	Practical year	∑ Students	<5 years medical practice	5-10 years medical practice	>10 years medical practice	∑ Physicians	Full sample
strong positive recommendation									
treat *all* eligible patients	161 (84.7)[a,c]	218 (94.8)[a,b]	109 (91.6)[b,c]	**488 (90.5)****	299 (89.8)[c]	170 (91.4)[b]	458 (81.5)[b,c]	**927 (85.8)****	1415 (87.3)
treat *selected* patients	13 (6.8)	5 (2.2)	2(1.7)	20 (3.7)	11 (3.3)	6 (3.2)	42 (7.5)	61 (5.5)	81 (4.9)
consider to treat	16 (8.4)	7 (3.0)	8 (6.7)	31 (5.8)	23 (6.9)	10 (5.4)	62 (11)	97 (8.8)	128 (7.8)
weak positive recommendation									
treat *all* eligible patients	18 (9.5)	15 (6.5)	12 (10.1)	45 (8.3)	34 (10.2)[a,c]	23 (12.4)[a,b]	38 (6.8)[b,c]	95 (8.8)	140 (8.6)
treat *selected* patients	87 (45.8)[a,c]	133 (57.8)[a,b]	65 (54.6)[b,c]	**285 (52.9)****	179 (53.8)[a,c]	80 (43)[a,b]	221 (39.3)[b,c]	**480 (44.4)****	765 (47.2)
consider to treat	85 (44.7)	82 (35.7)	42 (35.3)	**209 (38.8)****	120 (36)[a,c]	83 (44.6)[a,b]	303 (53.9)[b,c]	**506 (46.8)****	715 (44.1)
strong negative recommendation									
treat *none of* eligible patients	147 (77.4)[a,c]	200 (87.0)[a]	110 (92.4)[c]	**457 (84.8)****	304 (91.3)	169 (90.9)	505 (89.9)	**978 (90.5)****	1435 (88.6)
treat *selected* patients	13 (6.8)	4 (1.7)	4 (3.4)	21 (3.9)	6 (1.8)	7 (3.8)	15 (2.7)	28 (2.6)	49 (3.0)
consider to treat	30 (15.8)	26 (11.3)	5 (4.2)	61 (11.3)	23 (6.9)[a]	10 (5.4)[a]	42 (7.5)[a]	75 (6.9)*	136 (8.4)
weak negative recommendation									
treat *none of* eligible patients	58 (30.5)[c]	67 (29.1)[b]	54 (45.4)[b,c]	**179 (33.2)*****	132 (39.6)	83 (44.6)	261 (46.4)	**476 (44)*****	655 (40.4)
treat *selected* patients	44 (23.2)[a,c]	84 (36.5)[a,b]	33 (27.7)[b,c]	**161 (29.9)****	90 (27)[a,c]	49 (26.3)[a,b]	110 (19.6)[b,c]	**249 (23.0)****	410 (25.3)
consider to treat	88 (46.3)[a,c]	79 (34.3)[a]	32 (26.9)[c]	199 (36.9)	111 (33.3)	54 (29)	191 (34)	356 (32.9)	555 (34.3)

Note. Data are shown in N (%). All percentages refer to columns. Column differences (i.e. differences between subgroups) were examined using Chi^2-test followed by Bonferroni-corrected post-hoc Z- tests for column proportions (for exact values, see text)

*asterisks indicate significant differences between the subgroups of students and physicians, respectively (**p<.01, ***p<.001)

Equal letters ([aa, bb, cc]) indicate significant differences (p<.05) within the subgroups of students and physicians, respectively.

Supplementary Table 4: Distribution of proportions of respondents per type of recommendation, who felt unable to provide a quantitative estimate on the visual analog scale (ranging from 0 – 100 patients; survey part two) *Note*. Data are shown in N (%). All percentages refer to columns. Column differences (i.e. differences between subgroups) were examined using Chi^2-test followed by Bonferroni-corrected post-hoc Z- tests for column proportions (for exact values, see text)

No quantification (VAS)	Preclinical stage	Clinical stage	Practical year	∑ Students	<5 years medical practice	5-10 years medical practice	>10 years medical practice	∑ Physicians	Full sample
recommendation									
strong positive	57 (30.0)	48 (20.9)	24 (20.2)	**129 (23.9)*** **	36 (10.8)	25 (13.4)	68 (12.1)	**129 (11.9)*** **	258 (15.9)
weak positive	72 (37.9)	73 (31.7)	37 (31.1)	**182 (33.8)*** **	64 (19.2)	42 (22.6)	127 (22.6)	**233 (21.6)*** **	415 (25.6)
strong negative	60 (31.6)	53 (23.0)	24 (20.2)	**137 (25.4)*** **	44 (13.2)	32 (17.2)	53 (9.4)	**129 (11.9)*** **	266 (16.4)
weak negative	67 (35.3)	74 (32.2)	38 (31.9)	**179 (33.2)*** **	75 (22.5)	50 (26.9)	122 (21.7)	**247 (22.8)*** **	426 (26.3)

*asterisks indicate significant differences between the groups of students and physicians, respectively (***p<.001)

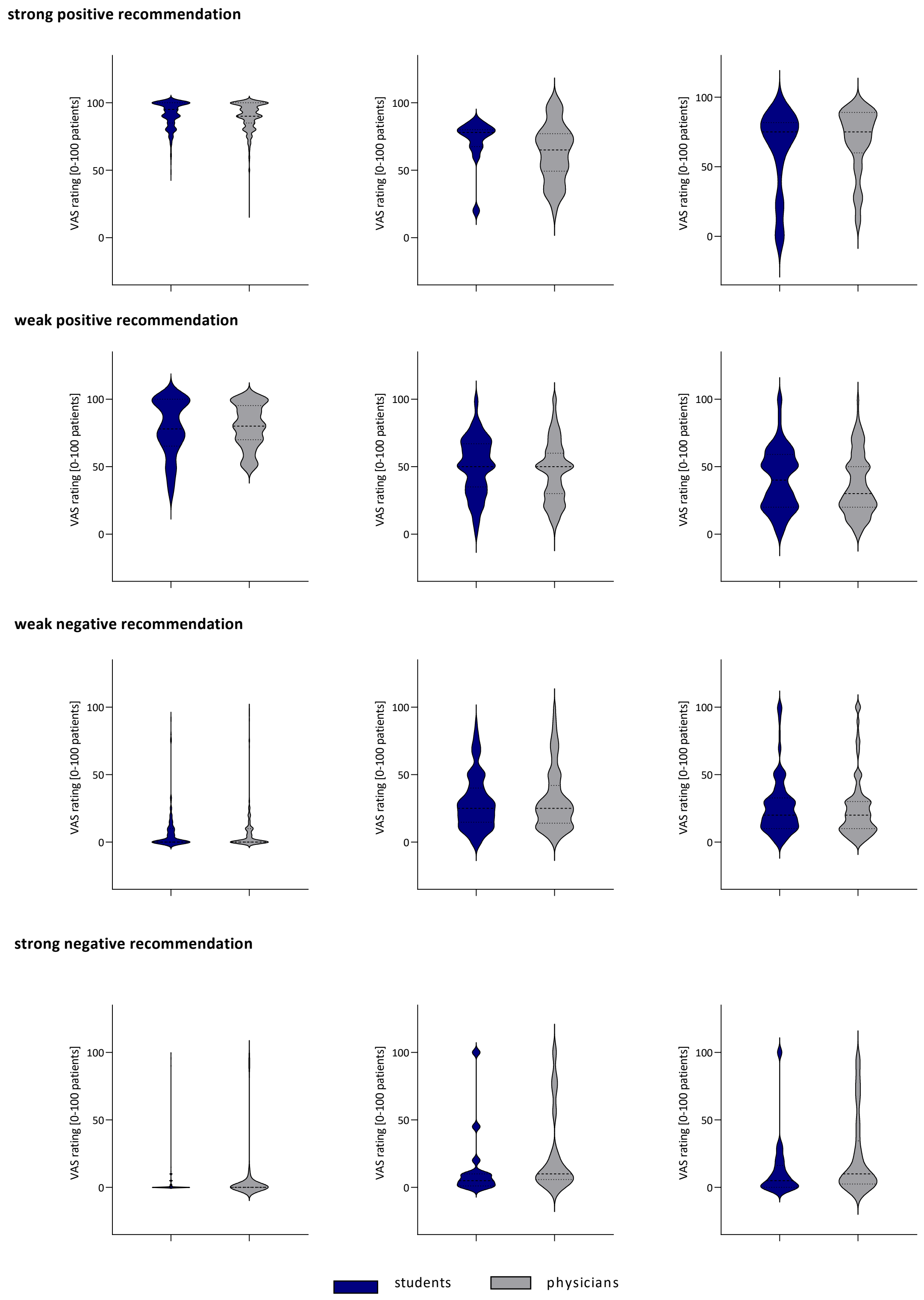

Supplementary Figure 1: Violin plots illustrate the distribution of answers from of digital sliding scale ratings for each of the four recommendations (survey section part 2b). Lines are plotted at the median (thicker lines) and the two quartiles (thinner lines). Data are shown separately for participants who indicated in survey section part 2a to apply the treatment in all or none of their patients (left panel), to apply the treatment in particular patients only (center panel), or to only consider the treatment for their patients (right panel). Students are indicated in blue, physicians in grey.

SURVEY (GERMAN ORIGINAL VERSION)

Sehr geehrte Studienteilnehmerin, Sehr geehrter Studienteilnehmer,

vielen Dank, dass Sie an unserer Umfrage teilnehmen.

Wir möchten untersuchen, wie die Empfehlungen, welche in Leitlinien zur Behandlung von Erkrankungen gegeben werden, von unterschiedlichen Zielgruppen interpretiert werden. Eine tiefergehende Kenntnis darüber, wie die sprachliche Ausformulierung solcher Leitlinienempfehlungen verstanden wird, ist wichtig, um klare und eindeutige Leitlinien zu entwickeln und ihre Umsetzbarkeit zu erleichtern.

Bitte beantworten Sie alle Fragen ehrlich und intuitiv. Auch wenn möglicherweise einige der Fragen ähnlich für Sie klingen, erfassen sie stets unterschiedliche Aspekte. Es ist daher wichtig, dass Sie alle Fragen beantworten.

Ihre Antworten werden anonym erfasst, ein Rückschluss auf Ihre Person ist nicht möglich. Die Teilnahme ist selbstverständlich freiwillig und Sie können die Bearbeitung jederzeit abbrechen. Die Daten werden am Institut für Medizinische Psychologie und Verhaltensimmunbiologie, Universitätsklinikum Essen verarbeitet und sollen in Kooperation mit der European Cardiology Section Foundation (Prof. Dr. Griebenow) veröffentlicht werden. Für die Studie liegt ein positives Votum der Ethikkommission der Medizinischen Fakultät der Universität Duisburg-Essen vor. Die Bearbeitung des Fragebogens nimmt weniger als 10 Minuten in Anspruch.

Bei Rückfragen stehen wir Ihnen gerne zur Verfügung: sven.benson@uk-essen.de

Vielen Dank für Ihre Unterstützung!

Prof. Dr. Sven Benson
Prof. Dr. Reinhard Griebenow
M. Sc. Justine Schmidt

A Bitte geben Sie Ihr Geschlecht an. ☐ weiblich ☐ männlich ☐ divers

B Sind Sie …?

Studierende/ Studierender im ersten Abschnitt der Ärztlichen Ausbildung (1.-4. Semester)................ ☐

Studierende/ Studierender im zweiten Abschnitt der Ärztlichen Ausbildung (5.-10. Semester) .... ☐

Studierende/ Studierender in der praktischen Ausbildung (Praktisches Jahr) .... ☐

Fachärztin/Facharzt für Allgemeinmedizin.. ☐

Fachärztin/Facharzt in einem Fachgebiet der Innere Medizin .... ☐

Fachärztin/Facharzt in einem Fachgebiet der Chirurgie .... ☐

Fachärztin/Facharzt für Frauenheilkunde und Geburtshilfe .... ☐

Fachärztin/Facharzt für Neurologie .... ☐

Fachärztin/Facharzt für Psychiatrie und Psychotherapie .... ☐

Fachärztin/Facharzt für Psychosomatische Medizin und Psychotherapie .... ☐

Andere Fachärztin/anderer Facharzt (wenn Sie sich aktuell in einer Facharzt-Weiterbildung befinden, tragen Sie bitte hier Ihre Fachrichtung mit dem Zusatz „in Weiterbildung zur/ zum…“ ein)):

____

C Falls Sie als Fachärztin/ Facharzt tätig sind, seit wie vielen Jahren üben Sie diese Tätigkeit aus?

seit weniger als 5 Jahre	seit 5 bis 10 Jahren	seit mehr als 10 Jahren
☐	☐	☐

Nachfolgend finden Sie Aussagen zur Wirksamkeit eines Medikaments

A Die Behandlung mit Medikament x geht einher mit einer Verbesserung der Lebenserwartung

B Die Behandlung mit Medikament x bewirkt eine Verbesserung der Lebenserwartung

C Die Behandlung mit Medikament x könnte eine Verbesserung der Lebenserwartung bewirken

Bitte bewerten Sie im Folgenden die Aussagen zur Wirksamkeit, die unter A, B und C getroffen wurden.

- ☐ A=B=C
- ☐ A=B>C
- ☐ A>B=C
- ☐ B>A>C
- ☐ B>A=C
- ☐ A>B>C
- ☐ C>B=A
- ☐ C>B>A

Eine Gruppe von Fachspezialist*innen bewertet regelmäßig medizinische Behandlungen auf Basis der wissenschaftlich ermittelten Behandlungseffekte. Die daraus abgeleiteten Empfehlungen für die ärztliche Praxis werden unterteilt in „stark positiv“, „schwach positiv“, „schwach negativ“ und „stark negativ“.

1a. Für den Fall einer **„stark positiven“** Empfehlung, wie setzen Sie diese in der Behandlung Ihrer Patient*innen um?

Ich werde die Behandlung bei allen in Frage kommenden Patient*innen anwenden ☐

Ich werde die Behandlung nur in besonders aussichtsreichen Fällen anwenden ☐

Ich werde die Behandlung bei in Frage kommenden Patient*innen lediglich in Erwägung ziehen ☐

1b. Von 100 für diese Behandlung in Frage kommenden Patient*innen, bei wie vielen würden Sie diese Behandlung anwenden?

Bitte geben Sie Ihre Einschätzung auf der folgenden Skala an, die von **0 (bei *keinem* der in Frage kommenden Patient*innen)** bis **100 (bei *allen* der in Frage kommenden Patient*innen)** reicht.

Wenn Sie diese Einschätzung nicht geben können, kreuzen Sie bitte weiter unten **„kann ich nicht beurteilen“** an.

0 ———————————————— 100

☐ Kann ich nicht beurteilen

2a. Für den Fall einer **„schwach positiven“** Empfehlung, wie setzen Sie diese in der Behandlung Ihrer Patient*innen um?

Ich werde die Behandlung bei allen in Frage kommenden Patient*innen anwenden ☐

Ich werde die Behandlung nur in besonders aussichtsreichen Fällen anwenden ☐

Ich werde die Behandlung bei in Frage kommenden Patient*innen lediglich in Erwägung ziehen ☐

2b. Von 100 für diese Behandlung in Frage kommenden Patient*innen, bei wie vielen würden Sie diese Behandlung anwenden?

Bitte geben Sie Ihre Einschätzung auf der folgenden Skala an, die von **0 (bei *keinem* der in Frage kommenden Patient*innen)** bis **100 (bei *allen* der in Frage kommenden Patient*innen)** reicht.

Wenn Sie diese Einschätzung nicht geben können, kreuzen Sie bitte weiter unten **„kann ich nicht beurteilen“** an.

0 ———————————————— 100

☐ Kann ich nicht beurteilen

3a. Für den Fall einer **„schwach negativen“** Empfehlung, wie setzen Sie diese in der Behandlung Ihrer Patient*innen um?

Ich werde die Behandlung bei keinem der in Frage kommenden Patient*innen anwenden ☐

Ich werde auf die Behandlung nur in besonders risikoreichen Fällen verzichten ☐

Ich werde die Behandlung bei allen in Frage kommenden Patient*innen in Erwägung ziehen ☐

3b. Von 100 für diese Behandlung in Frage kommenden Patient*innen, bei wie vielen würden Sie diese Behandlung anwenden?

Bitte geben Sie Ihre Einschätzung auf der folgenden Skala an, die von 0 **0 (bei *keinem* der in Frage kommenden Patient*innen)** bis **100 (bei *allen* der in Frage kommenden Patient*innen)** reicht.

Wenn Sie diese Einschätzung nicht geben können, kreuzen Sie bitte weiter unten **„kann ich nicht beurteilen“** an.

0 ———————————————————— 100

☐ Kann ich nicht beurteilen

4a. Für den Fall einer **„stark negativen“** Empfehlung, wie setzen Sie diese in der Behandlung Ihrer Patient*innen um?

Ich werde die Behandlung bei keinem der in Frage kommenden Patient*innen anwenden ☐

Ich werde auf die Behandlung nur in besonders risikoreichen Fällen verzichten ☐

Ich werde die Behandlung bei allen in Frage kommenden Patient*innen in Erwägung ziehen ☐

4b. Von 100 für diese Behandlung in Frage kommenden Patient*innen, bei wie vielen würden Sie diese Behandlung anwenden?

Bitte geben Sie Ihre Einschätzung auf der folgenden Skala an, die von 0 **0 (bei *keinem* der in Frage kommenden Patient*innen)** bis **100 (bei *allen* der in Frage kommenden Patient*innen)** reicht.

Wenn Sie diese Einschätzung nicht geben können, kreuzen Sie bitte weiter unten **„kann ich nicht beurteilen“** an.

0 ———————————————————— 100

☐ Kann ich nicht beurteilen

SURVEY (TRANSLATED VERSION)

Dear study participants,

Thank you very much for participating in this survey.

We wish to examine how the recommendations in the guidelines for treating diseases are interpreted by different target groups. A deeper understanding of how the wording of such guideline recommendations is received is important for developing clear and unambiguous guidelines and making their implementation easier.

Please answer all of the questions honestly and intuitively. Even if it seems to you that some of the questions are similar, they are indeed covering different aspects. It is therefore important that you answer all of the questions.

Your responses will be anonymously recorded; it will be impossible to identify you. Participation is voluntary and you may stop the survey at any time. The data will be processed at the Institute of Medical Psychology and Behavioral Immunobiology at the University Hospital Essen and are intended for publication in cooperation with the Cardiology Section Foundation (Prof. Dr. Griebenow). Approval has been given for this study by the Ethics Committee for the Medical Faculty of the University of Duisburg-Essen. It should take less than 10 minutes to fill out this survey.

Should you have any questions, please do not hesitate to ask us. We will be happy to help: sven.benson@uk-essen.de

Thank you for your support!

Prof. Dr. Sven Benson
Prof. Dr. Reinhard Griebenow
M. Sc. Justine Schmidt

A Please indicate your gender. ☐ female ☐ male ☐ other

B Which of the following describes you?

Student in the preclinical phase of medical education (1st-4th semester) ☐
Student in the clinical phase of medical education (5th-10th semester) ☐
Student in the practical training phase ("Practical Year") ☐
Medical Specialist in General Practice ☐
Medical Specialist in a field of Internal Medicine ☐
Medical Specialist in a field of Surgery ☐
Medical Specialist in Gynecology and Obstetrics ☐
Medical Specialist in Neurology ☐
Medical Specialist in Psychiatry and Psychotherapy ☐
Medical Specialist in Psychosomatic Medicine and Psychotherapy ☐
Other Medical Specialist (if you are currently undergoing specialist training, please state your discipline and "in training to become a (qualification sought)"):
____

C If you are a medical specialist, how many years have you practiced in this specialty?

less than 5 years	5-10 years	more than 10 years
☐	☐	☐

Here are three statements about the efficacy of a medication:

A Treatment with drug x is associated with improved life expectancy.

B Treatment with drug x causes improved life expectancy.

C Treatment with drug x could improve life expectancy.

Please select the best shorthand equation to reflect the statements about efficacy in A, B and C.

- ☐ A=B=C
- ☐ A=B>C
- ☐ A>B=C
- ☐ B>A>C
- ☐ B>A=C
- ☐ A>B>C
- ☐ C>B=A
- ☐ C>B>A

A group of medical specialists regularly evaluates medical treatment on the basis of scientifically determined treatment effects. The recommendations derived for medical practice are divided into the categories "strong positive," "weak positive," "weak negative," and "strong negative."

1a. In the case of a **"strong positive"** recommendation, how do you implement this in the treatment of your patients?

I use the treatment on all patients who are eligible for it. ☐

I use the treatment only in particularly promising cases. ☐

I only consider the treatment for patients who are eligible for it. ☐

1b. Out of 100 patients who are eligible for this treatment, how many patients would you treat?

Please estimate this number on the following scale from **0 (*none* of the eligible patients)** to **100 (*all* of the eligible patients)**.

If you are unable estimate this, please mark the box below "**unable to estimate**."

0 ———————————————— 100

☐ Unable to estimate

2a. In the case of a "**weak positive"** recommendation, how do you implement this in the treatment of your patients?

I use the treatment on all patients who are eligible for it. ☐

I use the treatment only in particularly promising cases. ☐

I only consider the treatment for patients who are eligible for it. ☐

2b. Out of 100 patients who are eligible for this treatment, how many patients would you treat?

Please estimate this number on the following scale from **0 (*none* of the eligible patients)** to **100 (*all* of the eligible patients)**.

If you are unable estimate this, please mark the box below "**unable to estimate**."

0 ———————————————— 100

☐ Unable to estimate

3a. In the case of a **"weak negative"** recommendation, how do you implement this in the treatment of your patients?

I use the treatment on none of the patients who are eligible for it. ☐

I refrain from treatment only in particularly high-risk cases. ☐

I consider the treatment for all eligible patients. ☐

3b. Out of 100 patients who are eligible for this treatment, how many patients would you treat?

Please estimate this number on the following scale from **0 (*none* of the eligible patients)** to **100 (*all* of the eligible patients)**.

If you are unable estimate this, please mark the box below "**unable to estimate**."

0 ____ 100

☐ Unable to estimate

4a. In the case of a **"strong negative"** recommendation, how do you implement this in the treatment of your patients?

I use the treatment on none of the patients who are eligible for it. ☐

I refrain from treatment only in particularly high-risk cases. ☐

I consider the treatment for all eligible patients. ☐

4b. Out of 100 patients who are eligible for this treatment, how many patients would you treat?

Please estimate this number on the following scale from **0 (*none* of the eligible patients)** to **100 (*all* of the eligible patients)**.

If you are unable estimate this, please mark the box below "**unable to estimate**."

0 ____ 100

☐ Unable to estimate
