# Supplementary material for: Evidence-informed language: interpretation and impact on intentions to treat – results of an online survey of medical students and specialists in German-speaking countries
Source: BMJ Open. 2025 Feb 7;15(2):e082907. doi: 10.1136/bmjopen-2023-082907 (PMC11808923; doi:10.1136/bmjopen-2023-082907)
Supplement: Supplementary file 2 [file bmjopen-2023-082907.reviewer_comments.pdf]

PEER REVIEW HISTORY

BMJ Open publishes all reviews undertaken for accepted manuscripts. Reviewers are asked to complete a checklist review form and are provided with free text boxes to elaborate on their assessment. These free text comments are reproduced below.

ARTICLE DETAILS

Title (Provisional)

Evidence-informed language: interpretation and impact on intentions to treat - Results of an online survey of medical students and specialists in German-speaking countries

Authors

Griebenow, Reinhard; Schmidt, Justine; Herrmann, Henrik; Benson, Sven

---

VERSION 1 - REVIEW

**Reviewer**	**1**
**Name**	**Maitland, Stuart**
**Affiliation**	**The Newcastle Upon Tyne NHS Hospitals Foundation Trust**
**Date**	**16-Jul-2024**
**COI**	**None declared**

The authors used an online survey to assess how medical students and doctors understood and complied with the description of different levels of evidence in their patient care. They asked respondents to rank strength of evidence for statements, and then how they would use specific evidence grade descriptors in clinical practice within categories and on a proportionate (percent of patients that they would offer treatment to) basis.

This is an interesting and important investigation in understanding how doctors and students use evidence and the descriptors that go with it. There are a number of areas that should be looked at to improve the strength of this article.

Abstract

L4 "while clinical recommendations require a clear framework to maintain the integrity of evidenced-based medical practice"- this is a redundant sentence that impedes the readibility of the abstract

L21- the structure of the section 1) is challenging to read. Please rephrase this using the terminology from the main methods section (e.g. "subjects ranked statements...")

L29- Please check editorial policy on number formatting however normally I would expect the BMJ to use commas as the delimiter for thousands.

Introduction:

P6L2- the first paragraph is confusing and does not add to the article. Please rewrite this

L7- As a computer scientist I implicitly understand the "doubling time of medical knowledge" but this may not be the case to many readers. Please rephrase or expand on this.

L10 "gained"- increased

Survey section 1:

P8- This has more importance in the results section, but consider referring to the recommendations options by a single shorthand name rather than a letter (as is done in table 2 "associated w, causes, could cause". Introducing these short titles here and using them in the results improves the readability of the manuscript as it means the reader isn't having to look back at what "A>B" means and can intuit "associated with > causes" as an incorrect statement.

L15- I disagree with the authors interpretation of the marburger bund proposal that C=A. The proposals seem to suggest that additional language should be used to separate randomised from non-randomised data, rather than they should be given equal weighting. The addition of these criteria makes it unclear what is considered a correct answer in the results- is this according to the HGJ editors, or the MB proposal, or both? I would therefore suggest to drop this standard, or explain how it is used in the assessment.

P9

L2-5- There are restricted options for the respondent to choose from. There is a world of difference away from "all eligible patients" and "only in promising cases". I would argue that there is no treatment that can be given in "all eligible patients" (perhaps not even CPR!) but that generally treatments that have strong evidence are applied in 95% of patients. A good example is how treatments in older individuals are deprescribed as their risk/benefit becomes uncertain and side-effects from polypharmacy become greater (e.g. https://doi.org/10.1093/ageing/afu145). I think that some discussion of the grey area between these first two options is warranted in the limitations as I think this is the biggest area of weakness of this paper.

P10- statistical power- how was this tested? Cohens?

P11- Table 1- Approbated is not a term used in English. This can be removed, physicians is clear enough as a descriptor

Gender- "Diverse"

L13- see earlier comments regarding substituting descriptors for grades rather than letters would improve readability here

L18- Please review statistical measures for multiple hypothesis testing and how these were used to amend limits for statistical significance (e.g. Bonferroni). This may have implications upon the results & discussion as to the areas that remain significant.

P15-16- the results are well-described statistically here with reference to tabular data. This also applied to P10 L17 where multiple hypotheses are tested without correction.

P23

L8-9- this is conjecture. I am unfamiliar with the German court system however in the UK we would generally use the principle of what a "majority of your peers" would do as the medical standard for negligence, and therefore a single expert witness would not testify as to what they would do, but what a body of their peers would do.

L11- "this study...and answers"- these two sentences are fragments and should be rewritten

P24- L7-9- this sentence can be removed as it is discussed elsewhere and doesn't belong in conclusions.

P38- Figure 1- please make the legends for the axes consistent with the description of the options, as in Table 2.

I feel that the violin plots are not as clear to understand as a simple boxplot, but I'd welcome if there's a reason the authors chose this method specifically.

Figure 2- This can be moved to supplementary files

P41- Suppl. Table 1- "approbated" as above

The table isn't quite clear what the two numbers refer to e.g. 284/49.

It's clear that general practice ("Family medicine") isn't represented in the study population. This is an important area to mention as a limitation as these doctors are often at the forefront of implementing policy decisions for public health e.g. Statins.

Reviewer 2

Name Gamakaranage, Champika

Affiliation Wrightington Wigan and Leigh NHS Foundation Trust, Gastroenterology

Date 26-Aug-2024

COI None

My main concern is the methodology and whether this research serves any clear clinical relevance in decision-making. Comparison of medical students to consultants doesn't seem to make a clear reason as to what we gain from it. This is not clear in the article.

VERSION 1 - AUTHOR RESPONSE

Manuscript ID bmjopen-2023-082907

Response to Reviewer #1

We thank Reviewer #1 for the thorough assessment of our manuscript, and we highly value the constructive and important considerations and remarks. Please find below detailed responses to all comments. In order to make the answers as clear and easy to understand as possible, the comments and answers are presented in tabular form.

Comment	Response
1. Abstract L4 "while clinical recommendations require a clear framework to maintain the integrity of evidenced-based medical practice"- this is a redundant sentence that impedes the readibility of the abstract	This sentence has been removed.
2. L21- the structure of the section 1) is challenging to read. Please rephrase this using the terminology from the main methods section (e.g. "subjects ranked statements...")	For the sake of clarity we have rewritten this paragraph
3. L29- Please check editorial policy on number formatting however normally I would expect the BMJ to use commas as the delimiter for thousands	Thank you for picking this up. We have corrected number formatting accordingly.
4. Introduction: P6L2- the first paragraph is confusing and does not add to the article. Please rewrite this	Thank you for your suggestion. Our aim in the first part of the introduction was to clarify that discussing the strengths of evidence and translation of evidence into language is essential to meet the ethical and professional requirements the medical profession has committed to worldwide. We are therefore grateful that you pointed out the lack of clarity and have rephrased this part accordingly.
5. L7- As a computer scientist I implicitly understand the "doubling time of medical knowledge" but this may not be the case to many readers. Please rephrase or expand on this.	We have rewritten this sentence.
6. L10 "gained"- increased	The sentence has been rephrased
7. P8- This has more importance in the results section, but consider referring to the recommendations options by a single shorthand name rather than a letter (as is done in table 2 "associated w, causes, could cause". Introducing these short titles here and using them in the results improves the readability of the manuscript as it means the reader isn't having to look back at what	We appreciate this suggestion. The respective sections have been rewritten. We now use shorthand names as suggested.

	"A>B" means and can intuit "associated with > causes" as an incorrect statement.	
8.	L15- I disagree with the authors interpretation of the marburger bund proposal that C=A. The proposals seem to suggest that additional language should be used to separate randomised from non-randomised data, rather than they should be given equal weighting. The addition of these criteria makes it unclear what is considered a correct answer in the results- is this according to the HGJ editors, or the MB proposal, or both? I would therefore suggest to drop this standard, or explain how it is used in the assessment.	We have intensively discussed the reviewer´s thoughtful comment. Following this comment, we have rewritten the paragraph as well as the corresponding part in the results section for the sake of clarification.
9.	L2-5- There are restricted options for the respondent to choose from. There is a world of difference away from "all eligible patients" and "only in promising cases". I would argue that there is no treatment that can be given in "all eligible patients" (perhaps not even CPR!) but that generally treatments that have strong evidence are applied in 95% of patients. A good example is how treatments in older individuals are deprescribed as their risk/benefit becomes uncertain and side-effects from polypharmacy become greater (e.g. https://doi.org/10.1093/ageing/afu145). I think that some discussion of the grey area between these first two options is warranted in the limitations as I think this is the biggest area of weakness of this paper.	We highly appreciate this comment including the reference, as it highlights the dilemma in making external recommendations without knowing the patient:<br>Current evidence does not suggest that increasing the number of graded recommendations is associated with acceptable discriminatory power (see references 21,22), and so, the paper cited by the reviewer also provides a wording suggesting that a given recommendation applies to "all" patients.<br>On the other hand, using a few simple recommendations (such as "all" patients) to ease decision-making has been criticized to offer too less differentiated options.<br>We have chosen to offer three options only: all patients; selected patients; no decision, but consideration only.<br>To clarify that "all" does not mean 100%, we have added "eligible", which at least excludes patients with contraindications.<br>Since the often-used term "selected" leaves open what the selection criteria are, we have explicitly offered the most likely mode of selection in everyday medical practice, i.e. choosing those patients with the highest likelihood for treatment success.<br>Therefore, we would leave this paragraph largely as it is, but now discuss this issue in the revised discussion/limitations section of the manuscript to address this important point.
10.	P10- statistical power- how was this tested? Cohens?	We have now added to the revised methods section that we used GPower Software to calculate statistical power:

	“Statistical tests were only performed in subgroups of >100 participants to ensure sufficient statistical power (i.e., 1-β > 0.90, α=0.05) for median effect sizes (d=0.5).”
11. P11- Table 1- Approbated is not a term used in English. This can be removed, physicians is clear enough as a descriptor<br>Gender- "Diverse"	Done as suggested.
12. L13- see earlier comments regarding substituting descriptors for grades rather than letters would improve readability here	Done as suggested.
13. L18- Please review statistical measures for multiple hypothesis testing and how these were used to amend limits for statistical significance (e.g. Bonferroni). This may have implications upon the results & discussion as to the areas that remain significant.	Many thanks for the comment. We have now clarified in the revised methods section / statistical methods section that we have corrected for multiple testing using the Bonferroni method. Please note that the results remain unchanged as we used Bonferroni correction already in the first version of the manuscript.
14. P15-16- the results are well-described statistically here with reference to tabular data. This also applied to P10 L17 where multiple hypotheses are tested without correction.	We highly appreciate the comment. Regarding Bonferroni correction please refer to comment 17
15. P23 L8-9- this is conjecture. I am unfamiliar with the German court system however in the UK we would generally use the principle of what a "majority of your peers" would do as the medical standard for negligence, and therefore a single expert witness would not testify as to what they would do, but what a body of their peers would do.	Thank you for pointing this out. This sentence has been changed accordingly.
16. L11- "this study...and answers"- these two sentences are fragments and should be rewritten	We have rewritten this paragraph.
17. P24- L7-9- this sentence can be removed as it is discussed elsewhere and doesn't belong in conclusions.	Thank you. We have rewritten this sentence in response to your comment and hope that we could clarify it´s role as a relevant part of the conclusion.
18. P38- Figure 1- please make the legends for the axes consistent with the description of the options, as in Table 2.<br>I feel that the violin plots are not as clear to understand as a simple boxplot, but I'd welcome if there's a reason the authors chose this method specifically.	We have changed the figure legends accordingly. We herein chose violin plots as they provide the same information as box plots (i.e., centre, spread, asymmetry and outliers), but offer density curves as an additional information. We think that illustrating density is herein important to support a better insight into the data distribution.
19. Figure 2- This can be moved to supplementary files	We agree to show this figure in the supplementary files and have adapted the results section accordingly.

20. P41- Suppl. Table 1- "approbated" as above The table isn't quite clear what the two numbers refer to e.g. 284/49. It's clear that general practice ("Family medicine") isn't represented in the study population. This is an important area to mention as a limitation as these doctors are often at the forefront of implementing policy decisions for public health e.g. Statins.	"Approbated" has been deleted. We clarified the meaning of the numbers (i.e. the number of specialists / trainees). We fully agree with the Reviewer that general practitioners are a highly relevant group. Please note that our sample included general practitioners (in the first version of the manuscript referred to "General Medicine", now changed to "General Medicine / Family Medicine"), even though in a comparatively low proportion of 6.5%. However, it must be considered that in Germany many specialists, especially internists, work as general practitioners, so that the proportion of doctors working in general medicine is probably underestimated here. We have taken up this aspect in the revised discussion.

Response to Reviewer #2

Comment:
My main concern is the methodology and whether this research serves any clear clinical relevance in decision-making. Comparison of medical students to consultants doesn't seem to make a clear reason as to what we gain from it. This is not clear in the article.

Response:
We thank Reviewer #2 for her critical comment. We have substantially revised the manuscript to address the comments of Reviewer #1 and hope that we have been able to clarify why this work contributes to our understanding of how to improve clinical decision-making. Current proposals for verbal differentiation of the strength of evidence (and in particular between randomized and non-randomized clinical trials) are publicly available and aim at all health professionals. Therefore, understanding of verbal evidence should ideally be tested in both professionals and students, as the formation of opinions about the role of evidence in clinical decision-making begins early in a medical career. Testing for differences between medical students and specialists may be helpful in detecting changes in opinion that occur at later stages of a medical career. However, comparing the responses of students and specialists was not the primary aim of this study, although these two groups represented the most likely samples for meaningful pre-specified subgroup analysis.
The main aim of the study was to determine whether differences in understanding and interpretation of verbal descriptors of the strength of evidence and clinical practice recommendations contribute to variability in clinical decision making, which has been widely documented in health services research studies.

VERSION 2 - REVIEW

**Reviewer**	**1**
**Name**	**Maitland, Stuart**
**Affiliation**	**The Newcastle Upon Tyne NHS Hospitals Foundation Trust**

**Date**	**27-Dec-2024**
**COI**	

-
